# Microenvironment and Tumor Heterogeneity as Pharmacological Targets in Precision Oncology

**DOI:** 10.3390/ph18060915

**Published:** 2025-06-18

**Authors:** Stelvio Tonello, Roberta Rolla, Paolo Amedeo Tillio, Pier Paolo Sainaghi, Donato Colangelo

**Affiliations:** 1 Dipartimento di Medicina Traslazionale, Università del Piemonte Orientale, Via Solaroli 17, 28100 Novara, Italy; pierpaolo.sainaghi@med.uniupo.it; 2 Dipartimento per lo Sviluppo Sostenibile e la Transizione Ecologica, Università del Piemonte Orientale, Piazza S. Eusebio 5, 13100 Vercelli, Italy; 3 Clinical Chemistry Laboratory, Maggiore della Carità Hospital, 28100 Novara, Italy; roberta.rolla@med.uniupo.it (R.R.); paoloamedeo.tillio@maggioreosp.novara.it (P.A.T.); 4 Dipartimento di Scienze della Salute, Farmacologia, Scuola di Medicina, Università del Piemonte Orientale, Via Solaroli 17, 28100 Novara, Italy

**Keywords:** precision medicine, intratumor heterogeneity, tumor microenvironment, molecular targeted, pharmacological therapy

## Abstract

Tumor diseases are characterized by high interindividual and intratumoral heterogeneity (ITH). The development and progression of neoplasms outline complex networks of extracellular and cellular signals that have yet to be fully elucidated. This narrative review provides a comprehensive overview of the literature related to the cellular and molecular mechanisms underlying the heterogeneity of the tumor mass. Furthermore, it examines the possible role of the tumor microenvironment in the development and support of the neoplasm, in order to highlight its potential in the construction of a diagnostic–therapeutic approach to precision medicine. Many authors underline the importance of the tumor microenvironment (TME) as it actively takes part in the growth of the neoplastic mass and in the formation of metastases and in the acquisition of resistance to anticancer drugs. In specific body districts, the ideal conditions occur for the TME establishment, particularly the inflammatory state, the recruitment of cell types, the release of specific cytokines and growth factors, hypoxic conditions. These components actively intervene by enabling tumor progression and construction of physical barriers shaped by the extracellular matrix that contribute to forming peripheral tolerance by intervention of myeloid precursors and the polarization of M2 macrophages. In recent years, ITH and the TME have assumed an important position in cancer research and pharmacology as they enable understanding the dense network of communication existing between the neoplasm and the surrounding environment, and to monitor and deepen the effects of drugs with a view to develop increasingly precise and effective therapies. In the last decade, knowledge of TME has been exploited to produce targeted molecular agents (inhibitory small molecules, monoclonal antibodies, gene therapy). Nonetheless, the bibliography shows the need to study ITH through new prognostic and predictive biomarkers (e.g., ctDNA and CTCs) and to increase its basic biology knowledge. Precision medicine is a new opportunity in the treatment of oncological diseases that is transforming the development of new drug approaches and their clinical use. Biology and biotechnologies are providing the bases for this revolution.

## 1. Introduction

Precision medicine in oncology describes therapeutic approaches that consider the molecular characteristics of a specific tumor and the microenvironment in which it develops, as well as distinctive traits of the patient, such as genetics and lifestyle. The goal of this strategy is to appoint personalized pharmacological treatment for the patient. This approach is significantly changing the diagnostic and therapeutic landscape of cancer as it can enable the overcoming of some of the major obstacles in oncology, such as drug resistance and genomic heterogeneity of tumors [1]. The monitoring of therapeutic response and drug interactions and the great scientific interest in this area are proved by the growing number of articles published [2,3,4]. Personalized medicine approaches patients considering their genetic characters with attention to their preferences, beliefs, attitudes, knowledge, and social context, while precision medicine describes a model for the delivery of healthcare that relies heavily on data, analysis and comprehensive information [5,6,7,8].

Solid tumors are characterized by the formation of a cell mass within a healthy tissue or organ. This set of cells constitutes the progeny of a somatic cell that has undergone a progressive series of aberrations in its genome (genetic mutations and/or epigenetic alterations) and is receiving the necessary stimuli to support and promote its proliferation [9]. The cell population is heterogeneous, as it is composed of different cell clones that differ in the phenotypic characteristics expressed. Each cell that characterizes the neoplastic mass can undergo further aberrations that may decide the acquisition of a replicative advantage in the microenvironment in which it develops compared to the others. Taken together, gene expression changes in one or more cells of the tumor mass are directly involved in changes in the biological behavior of the neoplasm [10,11,12,13]. The autonomous and progressive proliferation of neoplastic cells leads to the establishment of new relationships with the tissue elements of the tumor microenvironment. Thus, the microenvironment is crucial in tumor development and progression, and much scientific evidence seems to show some therapeutic potential related to its modulation [14,15,16,17].

The therapeutic approach of precision medicine is based on the molecular and individual characterization of the tumor, from the start of its onset. The identification of a precise pharmacological treatment by selecting the patient on the molecular characteristics of the neoplasm, could optimize the use of traditional cytotoxic therapies, thus improving the overall response and survival rate of patients. This approach is the next step to the classic “one-size-fits-all” approach, which involves the categorization of the tumor based on the primary site and histological type [12,18,19]. There are now national programs that are moving concretely towards a model linked to precision medicine [19,20,21].

Due to the heterogeneous tumor biology, it is often not enough to identify a single genetic alteration to obtain a target towards which to produce targeted therapies. In addition, the pharmacological modification of the intracellular signal networks of the neoplasm can decide the activation of alternative and/or compensatory pathways that can lead to a pharmacological modulation that is no longer effective than the target. This process, which involves therapeutic resistance, is linked to the cellular heterogeneity of the tumor and the tumor microenvironment [10]. Integration processes and information exchanges are therefore necessary to enable the application of precision medicine [22].

**Figure 1:**
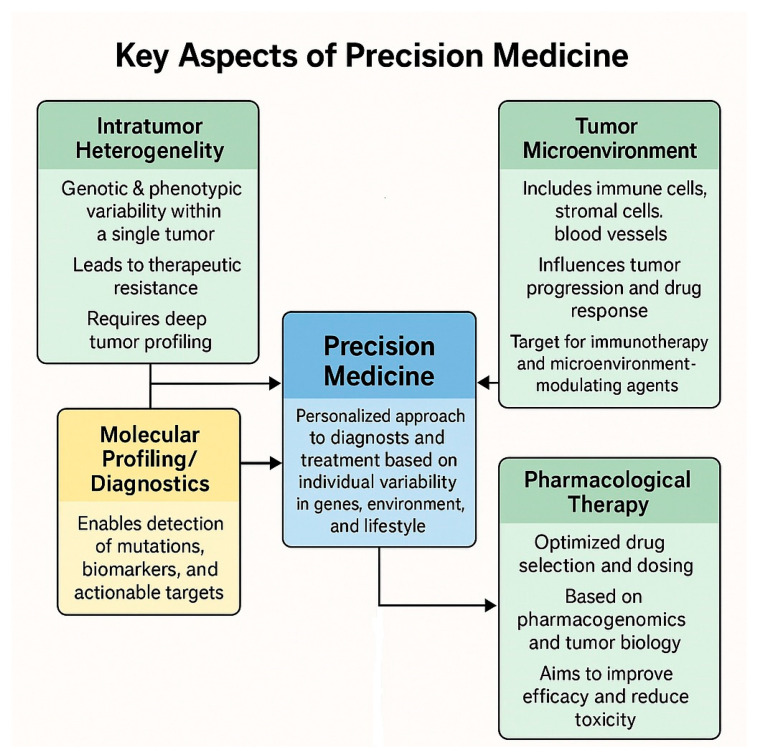
Schematic representation of the key aspects of precision medicine.

Recent advances in the field of “omics” are leading to the understanding of various molecular aspects of many diseases, including oncological ones. The result of the “multi-omics” analysis, which includes genomics, transcriptomics, epigenomics, proteomics, metabolomics, is beginning to have a concrete impact on the clinic and in the decision-making choice of oncological therapy, so much so that it is now possible to speak of “precision oncology” [5,23,24]. Precision medicine therefore is a new window on the biology of oncological diseases, and this is transforming the development of drugs and their clinical use (Figure 1).

## 2. The Tumor Microenvironment: A Complex Pharmacological Target for Precision Medicine

### 2.1. Tumor Heterogeneity

One of the most important aspects in the understanding of neoplastic development concerns the acquisition of replicative autonomy as it is often linked to the appearance of random mutations. “Driver mutations” are defined as genetic alterations that play a fundamental role in the initial stages of neoplastic development [25]. These mutations are associated with genomic regions responsible for regulating oncogenes involved in the control of cell proliferation. Many authors state that at least five driver mutations must coexist for the onset of the neoplastic cell to start from an altered somatic cell [25]. “Passenger mutations” are neutral or have minimal effects on cancer development. They accumulate because of genomic instability and high mutation rates during cancer cell development. These mutations generally affect tumor-suppressor genes and are secondary and accidental but can direct the etiology and evolution of the tumor [26,27].

Tumor heterogeneity occurs when the genome of different tumor clones dynamically evolves over time and accumulates persistent genetic mutations that will characterize subsequent subclones. The presence of tumor progenitor cells (CSCs) within the tumor microenvironment generates greater diversity as these cells are characterized by a variable spectrum of differentiation stages. These aspects give the newly formed cells phenotypic peculiarities (tumor heterogeneity) that can make them differ in their sensitivity to chemotherapy, radiotherapy, and targeted drug treatments. In addition, it is important to distinguish the intertumoral heterogeneity from the intratumoral heterogeneity (ITH). The first stands for all genomic, transcriptional, epigenomic, pathological and clinical differences that cause variation in therapeutic response between different patients with the same type of neoplasm. The ITH describes the multiple subclones that display different genomic, transcriptional, epigenomic and morphological characteristics [28,29,30,31,32]. These processes result in temporal heterogeneity, as the molecular characteristics of the subclonal structure vary over time also in relation to exposure to a hypothetical targeted treatment (therapeutic resistance). Furthermore, they have a spatial heterogeneity as tumor subclones show constitutive differences within the same primary tumor, or between the initial site and metastatic sites. In addition, a residual fraction of the heterogeneous mutation, even after invasive treatment, can remain in the body and cause a recurrence or reach the circulatory and/or lymphatic stream and be transported to sites distant from the primary one, acquiring metastatic capacity and making the pathology systemic [9,33,34]. Furthermore, ITH reduces the efficacy of therapeutic regimens and is often responsible for the resistance to chemotherapeutics, as the presence of multiple subclones also differentiates and modulates the therapeutic response. Therefore, ITH is closely related to tumor progression, therapeutic resistance, and recurrences [30,35]. These factors often make it difficult to find a therapy that works for every patient (the ideal “one size fits all”) while demanding a more complex approach that should be represented by precision medicine.

### 2.2. Intratumoral Heterogeneity (ITH): Clonal Evolutionary and the Cancer Stem Cell (CSCs) Models

To explain how intra-tumoral heterogeneity progresses over time, two exemplary models are reported, the clonal evolutionary model and the cancer stem cell (CSCs) model. In the clonal evolution model, all tumor cells derive from a single mutated cell (monoclonal origin) and acquire more driver mutations during tumor growth that confers heterogeneity. This can happen either in a linear fashion or through a branched mechanism. The first describes the emergence of a new mutation in the progeny that transmits a replication or survival advantage so powerful to surpass all previous clones. The branched mechanism gives rise to multiple populations of genetically different subclones as multiple clones can differentiate in parallel from a common ancestor following the acquisition of several driver mutations. The second model hypothesizes that only a few selected cancer cells have the potential to form new cancer cells, and it is the variability present within these that produces the heterogeneity seen in the tumor [9]. CSCs are self-renewable cells present in most of the primary sites of the tumor and are located within tumor niches, where they find favorable conditions for their implantation and survival following the activation of specific mechanisms (e.g., induction of angiogenesis, intervention of macrophages and stimulation of inflammatory processes, alterations of the extracellular matrix, and evasion of the defense systems of antitumor immunity) [9,15]. Mutations in normal stem cells handle the formation of multiple phenotypes of CSCs, increasing their proliferative ability, immune evasion, and reduction in apoptosis [36]. Experimental evidence suggests that the two models are not mutually exclusive, and that both may contribute to tumor heterogeneity [16]. The close link that coexists between tumor heterogeneity and evolution is fundamental in the development of neoplasia and can represent a valid starting point for prevention and precision therapeutic interventions. ITH can manifest morphological regions with varied differentiation, and molecular peculiarities through distinct gene expression patterns across tumor areas, often unrelated to clonal evolution [30]. Clonal and non-clonal ITH can be recognized. Clonal ITH transmits across generations, driven by mutations, CNAs, and epigenetic alterations. Mutations can be classified as ubiquitous, trunk, shared, or private, depending on their distribution across clones. Non-clonal ITH arises from microenvironmental influences leading to phenotypic plasticity via autocrine and paracrine signaling. Additionally, stochastic variations can create diversity at the single-cell level [36,37,38,39,40].

### 2.3. The Importance of the Tumor Microenvironment (TME)

Malignant tumors are complex structural entities that interact with the surrounding environment and create a network of connections capable of promoting the progression and metastasis of the neoplasm itself. The TME is, therefore, extremely important and is not an intrinsic feature driven by neoplastic cells but involves mechanisms of the cellular and extracellular environment that continuously take part in carcinogenesis and its heterogeneity [15,16].

As mentioned above, CSCs can unlimitedly differentiate into different cell types. The TME is characterized by the recruitment of specific cellular signals mediated by cytokines, chemokines, and growth factors. In addition, in recent years, the participation of other cell types existing in the TME has become increasingly clear, particularly the immune system cells, the endothelial cells and the stromal cells. In vivo tumor growth is influenced by a microenvironment characterized by extracellular matrix components, the partial pressure of oxygen (PO2) and small humoral molecules, such as cytokines. More interestingly, the TME is very complex and needs further investigations to be fully understood and to unveil its therapeutic target potential. The complex composition comprehends tumor cells, stromal cells-like mesenchymal stem cells (MSCs), vascular endothelial cells and pericytes, lymphatic cells, fibroblasts, myofibroblasts, immune system cells like macrophages, neutrophils, mast cells, NK cells, myeloid progenitors or MDSCs. Any of these cells seem to have a precise role in tumorigenesis [15,41,42,43,44].

### 2.4. Mesenchymal Stem Cells (MSCs)

MSCs derive from the stroma of the bone marrow and can differentiate into any tissue of mesenchymal origin (e.g., muscle, bone, tendons, ligaments, and adipose tissue). These cells have a high differentiation ability (plasticity). In addition, hypoxia present within the tumor niche stimulates the transcription and production of PGF, which promotes the homing of MSCs at the tumor site and the production of different chemokines (such as CXCL10 and CCL5) that have paracrine action on CSCs, promoting their progression and motility [45,46,47]. High levels of TNFs and IFN-gamma are present in tumor tissues increasing the expression of VCAM-1 on MSCs and activating their adhesion to endothelial cells, a process necessary for their migration into the tumor microenvironment [48,49,50].

A crucial role in tumor promotion supported by MSCs is played by TGF-beta. In late stages of the tumor, the increase in TGF-beta concentrations stimulates a process known as epithelial–mesenchymal transition (EMT), in which epithelial cells around the tumor mass change their morphology, becoming cells with the ability to migrate and initiate the process of metastasis [38,45,49,51]. This process is also mediated by metalloprotease 28 (MMP-28). TGF-beta also acts on immune cells by inhibiting the proliferation of NK cells and cytotoxic T-effector lymphocytes (CD8+). MSCs can inhibit the differentiation, maturation and function of mature dendritic cells involved in tumor antigen presentation and, therefore, impair the potential activation of CD8+. In addition, the presence of MSCs in the tumor microenvironment determines the increase in the concentration of IL-6 and IL-10. In particular, high levels of IL-10 are directly involved in the differentiation of M2-type tumor-associated macrophages (TAMs) which, in turn, secrete IL-10, increasing the anti-inflammatory process capable of bypassing activated immune defenses [52,53,54,55,56].

### 2.5. Tumor-Associated Fibroblasts (CAFs)

Fibroblasts are the cells that mainly make up connective tissue and have the main purpose of giving structure to tissues. Generally, activated fibroblasts have the function of suppressing tumor growth, but those associated with the tumor contribute positively to the course of tumorigenesis. Compared to normal fibroblasts, CAFs have higher proliferative activity, with high production of extracellular matrix and secretion of certain cytokines (e.g., SDF-1, VEGF, and PDGF) and extracellular vesicles [57,58,59]. Differences in fibroblast behavior and response result in tissue remodeling mediated by the expression of proteolytic enzymes (such as matrix metalloproteases, MMPs) and tumor angiogenesis, as pro-angiogenic factors (e.g., TGF-beta and PDGF) are released within the microenvironment. The heterogeneity that characterizes these cells can derive from the damage to which they have been exposed or from their origin, in any case there is a significant cellular plasticity that increases the stromal heterogeneity within the matrix and that makes CAFs clinically relevant in tumor understanding and development. For example, the abundance of stromal cells is related to a poor prognosis for several forms of cancer (e.g., breast and pancreatic cancer), and the high expression of matrix MMPs is related to increased neoplastic aggressiveness, as they are directly involved in the increase in metastatic invasiveness. Fibroblasts, myofibroblasts, pericytes and other cell types are often part of CAFs, although their function is partially understood [16,57,60].

### 2.6. Vascular and Immune Components of the TME

Angiogenesis plays a key role in tumor growth by supplying nutrients and oxygen to the neoplastic mass. Cancer stem cells (CSCs) recruit endothelial precursors, and endothelial cells support tumor self-renewal through autocrine cytokines. CSCs can circulate in blood and lymph as circulating tumor cells (CTCs), contributing to metastasis [14,16]. In hypoxic tumor areas, stabilization of HIF-1alpha activates gene expression programs, including VEGF-mediated neoangiogenesis—a major therapeutic target [35,61]. Elevated VEGF levels correlate with poor prognosis in several cancers. Tumor-associated macrophages (TAMs), recruited by CXCL12, also express HIF-1 alpha and contribute to angiogenesis and therapy resistance [62]. Immune cells are essential TME components, with early infiltration by innate immune cells followed by CD8+ activation via antigen-presenting cells [63]. However, regulatory T cells (Tregs), MDSCs, and immune checkpoints (e.g., CTLA-4 and PD-1) suppress effective antitumor responses [16]. Tumor vasculature modulates immune cell trafficking, and heterogeneous immune infiltration within lesions contributes to ITH and variable prognosis. TAMs, especially M2 subtype, dominate in solid tumors and are associated with poor outcomes [64]. M1 macrophages exert antitumor effects via IFN-gamma and pro-inflammatory cytokines, while M2 macrophages, induced by IL-4/10/13, support tumor growth [53,65,66].

## 3. Therapeutic Opportunities Offered by Precision Medicine

### 3.1. Precise Cancer Treatment Definition, Biomarkers, Targets, Types and Scope

The possible clinical application of precision medicine requires the presence of selective biomarkers of the neoplasm, able to provide indications on the type and progression of the disease itself. The term “biomarker” refers to a specific molecular alteration of the tumor that has a prognostic or predictive significance. A biomarker is prognostic when it can provide information on the progress of the disease, regardless of the therapies the patient receives. Instead, we refer to predictive factors when they provide information on the activity of specific therapies and enable the patient to be monitored based on the probability of responding or not to a given therapeutic agent. There are two types of predictive biomarkers: the first type enables the selection of patients who have a high probability of response to therapy (positive selection) and, generally, correspond to the molecular alterations that determine the constitutive activation of the molecular target of the drug. The second type of predictive biomarkers can identify patients who will not respond with high probability to a given therapy (negative selection) and correspond to mechanisms of resistance to the drug or to a class of drugs [67,68]. The level of tumor heterogeneity can also be a biomarker itself, differentiating the multiple subclones that characterize a particular neoplasm. In fact, advances in the detection and characterization of circulating tumor DNA (ctDNA) have also provided a valuable tool for individual genotyping of tumors and the identification of genetic and epigenetic alterations of the entire neoplasm to detect mutations associated with therapeutic resistance [69,70]. As mentioned above, there is continuous dialogue between cancer cells and the surrounding microenvironment mediated by stromal components, signaling factors (e.g., cytokines, chemokines, and growth factors) and other cell types. Therefore, it is clear that the tumor niche shapes the responses of the selective pressure exerted by drug treatment and can influence the emergence of resistant subclones [14,71]. To successfully implement precision therapeutic strategies, it is necessary to consider those that are currently widely used in clinical oncology. In fact, many FDA- and EMA-approved targeted therapies have proven remarkable clinical success in the treatment of cancer. The targets selected to produce drugs can be directed against the intrinsic peculiarity of the neoplastic cell (e.g., the possibility of inhibiting apoptosis) or against the tumor microenvironment [72,73,74,75]. Initially, the concept of precision medicine in oncology has been used to describe the molecular approach against cancer, with treatments capable of inhibiting the function of a molecular alteration. To date, this term is also associated with immunotherapy and therapeutic agents targeting any mutation associated with tumorigenesis [8,17,76]. The available precise drug treatments that fall under the classification are inhibitory small molecules, monoclonal antibodies, and gene therapy. Inhibitory small molecules are antagonists that have a relatively low molecular weight (<900 Da) and are capable of penetrating cells to target specific intracellular proteins. Many drugs are antagonists of kinases associated with TK-receptors and to the signaling pathways dysregulated during tumorigenesis. These drugs can be used to act on proteasomes, cyclin kinases and-(CDK) and poly inhibitors-ADP ribose polymerase (PARP) capable of activating the cell cycle checkpoints, triggering apoptosis, and modulating DNA repair [77,78]. Examples of small molecule inhibitors treated are listed in Table 1.

One of the key mechanisms in precision therapy is related to the induction of apoptosis of cancer cells. Apoptosis, known as programmed cell death, is a physiological process that is responsible for eliminating damaged or abnormal cells to keep the body’s homeostasis. However, the control of apoptosis by the neoplasm plays a fundamental role in promoting tumorigenesis and overexpression of Bcl-2 in some lymphoid malignancies has been proven important for proliferation and linked to increased resistance to chemotherapy. An example of anti-BCL-2 precise treatment is the drug venetoclax since it blocks the mitochondrial anti-apoptotic B-cell lymphoma-2 (Bcl-2) protein, leading to programmed cell death of CLL cells or small lymphocytic lymphoma (SLL) [79,80].

Monoclonal antibodies (mAbs), produced by hybridoma technologies, selectively bind extracellular proteins and inhibit tumor growth by disrupting the interaction between receptors and their endogenous ligands. They can mediate their action through selective direct binding of antigens on the target cell (receptor or ligand), or indirect binding, through activation of immune defenses following the recruitment of effector immune cells and phagocytes. This approach is often used in immunotherapy to trigger the immune system to attack cancer cells, by activating different processes like Antibody Cellular Cytotoxicity (ADCC), Antibody Cellular Phagocytosis (ADCP), Complementary Cytotoxicity Dependent (CDC), Signal Transduction Blocking (STB), or induction of apoptosis. Pharmacology is offering a wide variety of new bi- or multispecific antibodies and antibody-drug conjugates (ADC) that are going to dramatically improve the therapy in cancer and other diseases [81,82]. ADCs are extremely selective and enable specific cytotoxic action towards the tumor selective target. Some of the most effective approved by the FDA are Brentuximab Vedotin for Hodgkin’s lymphoma and anaplastic large cell lymphoma, ado-trastuzumab emtansine for HER2-positive metastatic breast cancer and inotuzumab ozogamicin for specific types of acute lymphoblastic leukemia (ALL) in adults and children. Some important mAbs that are making the difference in cancer therapy in the last decade are listed in Table 2.

Monoclonal antibodies in combination with other agents have been suggested for the inhibition of specific cancer pathways, like for example the Notch pathway. This receptor signaling modulates tumor cell growth, especially in colorectal cancer (CRC), and is involved in the immune landscape within the tumor microenvironment (TME). In fact, aberrant Notch signaling in CRC promotes immune evasion and tumor progression through modulation of immune cells functions in its microenvironment, especially myeloid-derived suppressor cells (MDSCs) and TAMs [63,68,71,72,73,74,75,76,77,78,79,80,81,82,83,84,85].

A completely different approach to precision medicine is offered by gene therapy. This pharmacological approach aims to introduce genetic material into cancer cells to inhibit their growth and likely induce death. Nowadays, there are multiple approaches that have gathered interesting clinical results:

- Replacement of the mutated tumor-suppressor gene with a normal gene to restore its function,
- Inhibition of the expression of one specific oncogene,
- Stimulation of the immune response,
- Inhibition of angiogenetic processes,
- Sensitization of cancer cells to other cancer treatments, and
- Modulation of the TME.

Methods used for gene therapy include RNA interference, DNA and RNA vaccination, miRNA applications and transfection of specific gene portions. The introduction of the gene can be made in vivo by viral vectors (e.g., by adenoviruses and lentiviruses) or non-viral vectors (mainly liposomes or nanovectors) [55,86]. Ex vivo methods can be performed by removing the target cells from cancer mass or the immune system cell from blood, transferring the gene in vitro and following amplification of the clone, inoculating it into back to the patient. An example of this approach is CRISPR-Cas9 gene editing, which is one of the most important editing tools used in gene therapy. This technology enables us to edit the genes involved in the development and progression of tumors by correcting mutations or the inactivation of oncogenes, as well as the modification of pharmacological and resistance targets. In general, CRISPR (clustered regularly interspaced short palindromic repeats) is transcribed into a short RNA sequence that acts as a guide (sgRNA) and is selectively directed towards the target gene sequence. Subsequently, Cas9, an endonuclease, binds to the target DNA sequences bound to sgRNA and silence it. This technology has been used to inhibit the gene encoding PD-1 and, therefore, to enable the intervention of CD8+ with antitumor action in multiple myelomas, melanomas, and sarcomas and for modulating TME activity [87,88,89,90].

### 3.2. Tumor Microenvironment Targets

Drugs targeting cellular and extracellular components that characterize the tumor microenvironment have remarkably high potential in reducing tumor progression. A clinical example is the small molecule bindarit, used against melanoma, prostate, and breast cancer. Bindarit is a selective inhibitor of the monocyte chemotactic proteins (MCP) MCP-1/CCL2, MCP-3/CCL7, and MCP-2/CCL8. This molecule has anti-inflammatory properties and can abolish the recruitment of distinct types of leukocytes following inflammatory processes. The effects on the TME are related to the decrease in monocyte tumor infiltration and proliferation supported by TAMs and MDSCs and a reduction in tumor vascularization [91,92,93].

Another example is the immunostimulant drug plerixafor which inhibits CXCR4. This molecule is the ligand of the chemokine CXCL12, directly involved in the recruitment and renewal of CSCs, but also in the remodeling of the tumor microenvironment. The drug enables the reduction in the pool of CSCs and together with other agents seems to offer interesting developments in the modulation of the TME [94,95].

Since tumor proliferation and metastasis are highly dependent on the formation of the vascular component, antiangiogenic drugs that affects the TME have been clinically proposed. These drugs targets factors that stimulate blood vessel growth and plasticity, such as VEGF, TGF-beta, PDGF, MMPs and various growth factors. Drugs such as bevacizumab, sunitinib, sorafenib, and pazopanib block the activity of VEGF or its receptor (VEGR-R), causing steric clutter and blocking the signal transduction pathway [96,97,98,99].

The role of matrix metalloproteinase (MMPs), especially MMP2 and MMP9 (gelatinases) in cancer treatment, has been investigated for many years. MMPs are both targets to avoid tumor progression and metastases invasion and to increase the permeability of several factors within the tumor niche since they degrade the extracellular matrix. In fact, the microenvironment produces a barrier difficult to overcome, making the passage of nutrients and growth factors inaccessible for the innermost cellular component [71,100]. The diffusion and the multiple physiological functions of gelatinases make a precision treatment approach difficult. There are many agents that have been proposed to direct inhibit MMP2 and MMP9, although the indirect modulation of its transcription is offering better chances in therapy. Prednisolone inhibits monocyte differentiation and clodronate are also able to reduce MMP9 activity. These drugs have also the advantage to reduce both the immunosuppressive action of M2, thus slowing tumor growth, and the antitumor reactivation of M1 [71]. Gefitinib has been described able to decrease synthesis of matrix metalloproteinase of colon cancer cells, and more recently miRNA-124 showed to indirect inhibit MMP9 via the transcription factor Signal transducer and activator of transcription 3 (STAT3) modulation [101,102].

CAFs have been exploited for TME modulation [57,58]. An interesting approach proves that the transfection of a plasmid encoding secretable TNF-related apoptosis-inducing ligand (sTRAIL) in fibroblasts present in the microenvironment can block tumor growth [103,104].

TAMs can also be used to achieve an effective result in precision medicine. TAMs can bypass tumor defenses and reach the necrotic core of the tumor, thus enabling the targeted action of the drug associated with them [71]. Some Authors have described the possibility of switching the polarization of M2 to M1 using TLR agonists or drugs like trabectedin and lurbinectedin to induce cancer cell death modifying the stroma cells of the tumoral microenvironment and promoting the switch towards a pro-inflammatory polarization of macrophages [105,106,107].

Modulation of the immune system towards immunosuppressants and Tregs also plays an effective role in precision therapies. For example, immune checkpoint inhibitors like the drug ipilimumab, that blocks the binding between CD80 and CD86 ligands with the CD8+-associated CTLA-4 receptor, and nivolumab, that occupies the binding site of the PD-1 and does not enable the recognition of its PDL-1 ligand that turns off the immune response, might integrate more selective TME approaches. However, such strategies are non-specific and may be associated with the development of autoimmune diseases [108,109].

### 3.3. Liquid Biopsy

Some anticancer drugs, including molecular therapeutic agents and immunotherapeutics, have been shown to limited in their efficacy by the extensive survival of subclones that acquire the ability to resist treatment. Therefore, it is necessary to integrate the morphological and molecular characteristics of ITH to improve patient classification and response to therapy. For this reason, ctDNA might represent a predictive biomarker, capable of monitoring the action of precision pharmacological treatments. Liquid biopsy like peripheral blood and biological fluids (such as cerebrospinal fluid or urine), together with the molecular profiling of circulating free nucleic acids (cfDNA), could represent useful tools for obtaining a more complete clinical picture of the molecular evolution of the tumor, including ITH and the TME. In plasma, most of the cfDNA comes from leukocytes and a small part (represented by ctDNA) comes from cancer cells. The presence of circulating tumor DNA is linked to tumor cell death following necrosis and/or apoptosis [69]. In addition to ctDNA, liquid biopsies can also provide information about circulating tumor cells (CTCs). This investigation can provide both a qualitative real-time assessment of genomic instability between the different tumor niches, and a quantitative analysis of the tumor mass, as ctDNA and CTCs can come from different and heterogeneous metastatic sites [30]. The ctDNA content of peripheral blood can represent tumor heterogeneity more comprehensively than the DNA extracted from tissue biopsy. However, it provides little information on the representativeness of the sample or the cellularity from which it originates as it has a low detection rate. This can easily be overcome with new diagnostic and molecular profiling approaches of higher sensitivity, such as digital PCR (ddPCR) or NGS [39,70]. Biotechnology is enabling a quantum leap never seen before.

### 3.4. Critical Issues and Limitations in Actual Precise Medicine Strategies

The integration of the molecular characteristics of a tumor, the characteristics of the microenvironment in which it develops, the genetics and lifestyle of the patient new model of medicine can provide the basis for rational health care in which the patient is the fulcrum and for the development of new drugs [110,111,112,113,114,115,116]. Many Authors stressed well the involvement of other cell types that compose the TME, such as some cells of the immune system, endothelial cells, and tumor-associated stromal and fibroblast cells (CAFs). Proteomics and in general all “omics” approaches, e.g., the systematic characterization of a given tissue, can be exploited to identify the resistant tumor phenotype and to compare this profile with that of sensitive tumor tissues, before starting treatments. The rational integration of many therapeutic agents can increase the effectiveness of individual treatments. This is the case of gefitinib and vandetanib which function as inhibitors of P-glycoprotein, a drug efflux pump used by cancer cells to induce therapeutic resistance in breast cancer, or monomethyl auristatin E (MMAE) conjugated with rituximab that has shown better efficacy than the single activity of rituximab in the treatment of diffuse large B-cell lymphoma, proving the efficacy of ADCs, also in association with CHOP chemotherapy (cyclophosphamide, doxorubicin, vincristine, prednisolone) [117,118]. However, side effects associated with the use of molecular therapies are still many and often associated both with the complexity of the tumor regulatory mechanisms and with the lack of selective biomarkers that do not enable therapy to be directed exclusively at neoplastic cells. Some examples of these adverse effects are skin rash, diarrhea, hypothyroidism, proteinuria, depigmentation, and hepatotoxicity [119]. These adverse drug reactions (ADRs) can be classified as off-target effects, likely caused by the binding of the drug to an unwanted target or the target present in other tissues where it exerts physiological activities. VEGF inhibition by bevacizumab has been shown to cause gastrointestinal perforation, complications during wound healing, bleeding, arterial and venous thromboembolism, proteinuria, and hypertension. Toxicity can also be influenced by the genetic predisposition of each individual which affects metabolism and response to drugs, but this field needs yet to be fully elucidated. In the last two decades many efforts have been made to reduce ADRs and improve the delivery of drugs. Nanovectors, magnetic nanoparticles and functionalized nanoparticles have some opportunities, among others, to overcome these issues [120,121,122,123]. In any case, ITH is still the greatest challenge in precision medicine, associated with both the discovery of specific tumor biomarkers and the evolution of increasingly precise therapies. The molecular profiling of tissue obtained from the individual tumor lesion is often not representative of systemic pathology. As a result, drugs directed against the identified molecular targets may not be effective in all metastatic tumor lesions, but therapeutic expectations in this area are extremely high.

Some critical issues still return in precise medicine, like resistance and TME adaptation to drug stress [124,125,126]. New insights on the TME and its possible remodeling for cancer treatment have been proposed, like extracellular vesicles analysis and engineering, but there is still much work to be done in this field [127,128,129]. Although there is still a lack of basic biological data on which more efforts should be focused, the road to precision medicine is now mapped out.

### 3.5. Translational Insights and Emerging Therapeutic Technologies: From Bench-to-Bedside

The TME has provided multiple insights into translational approaches for precise oncology. Several factors influence therapeutic outcomes; therefore, most reliable in vivo data come from animal studies. Some Authors have described how the aging of the tumor microenvironment and immune system can influence the TME composition, especially that related to the cellular composition of the immune system [130]. The immune system of elderly patients presents lower proportions of naive cytotoxic cells (TcN) and T helper cells (ThN), B cells and double-negative T cells. Therefore, the TME composition is affected and the efficacy of drugs such as immune checkpoint inhibitors (ICIs) may vary as a function of the age of the patients. Furthermore, some cellular and molecular aspects influence the response to immunotherapy and resistance to target molecules. Some important data come from clinical trials on several solid tumors, such as melanoma, lung, liver, renal and colon carcinoma. A key factor appears to be the number of tumor-infiltrating lymphocytes (TILs) expressing programmed cell death type 1 (PD-1), lymphocyte activation gene 3 (LAG3), T-cell immunoreceptor with Ig and ITIM domains (TIGIT), and T-cell immunoglobulin and mucin domain 3 (TIM3) markers. In these studies, drugs such as nivolumab and avelumab in combination with axitinib were responsible for a better hazard ratio of progression-free survival (a predictor of overall survival) [131]. Tumor-infiltrating lymphocytes (TILs) are an important component of the TME and may modulate resistance to PD-1/PD-L1 inhibitors. Indeed, lymphocyte activation gene 3 (LAG3), T-cell immunoreceptor with Ig and ITIM domains (TIGIT), and T-cell immunoglobulin and mucin domain 3 (TIM3) are linked to resistance and clonal selection in lung adenocarcinoma [114,131]. Regarding prevention and treatment of metastasis, many clinical trials have targeted TME features in advanced stages by arming NK cells with chimeric antigen receptors (CAR) (CAR-NK) and these approaches have shown significant contributions in immunotherapy, enabling the targeting of cell surface antigens directly to the TME. The dynamics of tumor infiltration and adaptation to the tumor microenvironment play a critical role in the efficacy of CAR-NK cells.

The combination of CAR-NK cell therapies with chemotherapy, radiotherapy, immune checkpoint inhibitors and epigenetic modulators is providing interesting clinical results. There are several antigenic targets for CAR T cells that are being investigated in clinical trials, with the most promising being EGFR, HER2, CEA and many others [132,133]. CRISPR gene editing has also been described to enhance CAR T cell activity, such as deletion of genes encoding TCR and inactivation of PD-1.

Vascular endothelial growth factor (VEGF) receptor tyrosine kinase inhibitors can modify the characteristics of the TME and have been widely used for the treatment of advanced metastatic renal cell carcinoma (RCC). Long-term survival with this treatment is poor, so recently ICI-based combination regimens such as nivolumab plus ipilimumab or ICI plus kinase inhibitors (pembrolizumab plus avelumab or lenvatinib) have become the standard of care in this tumor type. These drug combinations directly modify the TME, thus showing a clear clinical utility of TME-modulating drugs [134,135].

Recent approaches have guided the translation of oncology nanomedicines from the laboratory to the bedside. Clinical contributions are yet to be defined, but this approach is indicated to overcome the poor tumor diffusion of active drugs in the TME and for precise drug release. The peculiarities of the TME, such as weak acidity, high reactivity of oxygen species and hypoxia, have been exploited for modulation of TME permeability and activation of antiproliferative groups. These TME-activated nano-approaches appear to be able to influence drug activity, such as ICI or TK inhibitors, and the clinical possibilities are indeed broad [136,137,138,139]. In recent years, several strategies to dysregulate the bone marrow microenvironment have been described. TME modification in acute leukemia and neuroblastoma by extracellular vesicles (EVs) carrying micro-RNA (miRNA), siRNA and other active agents has been described as a potential strategy to reduce the spread of metastases and support tumor growth and preliminary in vivo results are promising [140,141]. Furthermore, multifunctional platforms have been subjected to clinical trials, such as CRISPR-based nanoparticles, which are in clinical and preclinical trials for triple-negative breast cancer [142].

## 4. Conclusions

The identification of increasingly effective prognostic and predictive tumor biomarkers and the development of innovative technologies used in diagnostics will continue to provide more comprehensive and precise information on the tumor complexity of each patient. Neoplasm genotyping and other integrated approaches will guide the best therapeutic choice for each patient and enable always improving the survival of cancer patients and their quality of life. The transition from the “one-size-fits-all” approach to precision medicine is particularly important in oncology as these diseases are characterized by a continuous genetic and environmental interaction unique to each patient. Future therapeutic approaches and disease prevention strategies will increasingly consider the individual variability in genes, environment, and lifestyles of each patient.

## Figures and Tables

**Table 1:** This table lists some relevant small molecule inhibitors commonly used in therapy, their mechanisms of action and main clinical applications.

Name of the Active Principle (Drug)	Mechanism of Action	Clinical Applications
Imatinib	BCR-ABL tyrosine kinase (TK) inhibitor	Treatment of chronic myeloid leukemia and gastrointestinal stromal tumors
Sorafenib	Multiple TK inhibitor	Treatment of unresectable liver carcinoma, advanced renal carcinoma, and differentiated thyroid carcinoma
Gefitinib	Blocks epidermal growth factor receptor TK	Treatment of non-small-cell lung cancer NSCLC
Vandetanib	Selective inhibitor of RET kinase	Treatment against the growth and spread of cancers related to the activation of RET oncogene, like medullary thyroid cancer and some NSCLC

**Table 2:** This table illustrates some examples of relevant mAbs routinely used in therapy, their mechanisms of action and main clinical applications.

Name of the Active Principle (Drug)	Mechanism of Action	Clinical Applications
Nivolumab and pembrolizumab	Bind and block programmed death-1 (PD-1) protein on T lymphocytes	Treatment of melanoma, non-small-cell lung cancer (NSCLC), kidney cancer, Hodgkin’s lymphoma, malignant pleural mesothelioma, bladder cancer, esophageal and stomach cancer
Cetuximab	Blocks the epidermal growth factor receptor (EGFR)	Treatment of metastatic colorectal and head and neck cancers that have a normal KRAS gene
Panitumumab	Blocks the epidermal growth factor receptor	Treatment of colorectal cancers that have a normal KRAS gene

## Data Availability

The authors agree that this manuscript will be shared with other qualified investigators through academically established means. This manuscript will be available on the public free “Università del Piemonte Orientale” institutional web page (https://research.uniupo.it/en/), and Linkedln Authors’ web pages.

## Abbreviations

The following abbreviations are used in this manuscript:

ADC	Antibody–Drug Conjugates
ADCC	Antibody–Dependent Cell-mediated Cytotoxicity
ADCP	Antibody–Dependent Cellular Phagocytosis
APC	Antigen-Presenting Cell
CAF	Cancer-Associated Fibroblast
CDC	Complement-Dependent Cytotoxicity
CAN	Copy Number Alteration
CRISPR	Clustered Regularly Interspaced Short Palindromic Repeats
CSC	Cancer Stem Cell
CTC	Circulating Tumor Cell
cfDNA	Circulating cell-free DNA
ctDNA	circulating tumor DNA
EMA	European Medicines Agency
EMT	Epithelial–Mesenchymal Transition
FDA	Food and Drug Administration
HIF	Hypoxia-Inducible Factor 1
HRE	Hypoxia-Responsive Elements
IFN	Interferon
THE	Interleukin
ITH	Intratumor Heterogeneity
mAb	Monoclonal Antibody
MMP	Matrix Metalloproteinase
MDSC	Myeloid-Derived Suppressor Cell
MSC	Mesenchymal Stem Cell
NGS	Next Generation Sequencing
NFkB	Nuclear Factor kappa B
NK	Natural Killer
PDGF	Platelet-Derived Growth Factor
PGF	Placental Growth Factor
SDF-1	Stromal Cell-Derived Factor 1
TAM	Tumor-Associated Macrophage
TGF	Tumor Growth Factor
TLR	Toll-Like Receptor
TME	Tumor Microenvironment
TNF	Tumor Necrosis Factor
Treg	T regulatory cell
VCAM-1	Vascular Cellular Adhesion Molecule-1
VEGF	Vascular Endothelial Growth Factor
